# A unique Smith-Magenis patient with a de novo intragenic deletion on the maternally inherited overexpressed *RAI1* allele

**DOI:** 10.1038/s41431-022-01143-5

**Published:** 2022-07-11

**Authors:** Alessandra Sironi, Ilaria Bestetti, Maura Masciadri, Francesca Tumiatti, Milena Crippa, Chiara Pantaleoni, Silvia Russo, Stefano D’Arrigo, Donatella Milani, Lidia Larizza, Palma Finelli

**Affiliations:** 1 Experimental Research Laboratory of Medical Cytogenetics and Molecular Genetics, IRCCS Istituto Auxologico Italiano, Milan, Italy; 2 Department of Medical Biotechnology and Translational Medicine, University of Milan, Milan, Italy; 3 Department of Development Neurology, Fondazione IRCCS Istituto Neurologico Carlo Besta, Milan, Italy; 4 Pediatric Highly Intensive Care, Medical Genetics Unit, Fondazione IRCCS Ca’ Granda Ospedale Maggiore Policlinico, Milan, Italy

**Keywords:** Gene expression, Neurodevelopmental disorders, Genetic testing

## Abstract

*RAI1* is a dosage-sensitive gene whose decreased or increased expression by recurrent and non-recurrent 17p11.2 deletions or duplications causes Smith-Magenis (SMS) or Potocki-Lupski syndromes (PTLS), respectively. Here we report on a 21-year-old female patient showing SMS phenotype who was found to carry a 3.4 kb de novo intragenic *RAI1* deletion. Interestingly, a significant increase in *RAI1* transcript levels was identified in the patient’s, brother’s and mother’s peripheral blood cells. Allele-specific dosage analysis revealed that the patient’s maternally inherited overexpressed *RAI1* allele harbors the intragenic deletion, confirming the SMS diagnosis due to the presence of a single wild-type *RAI1* functional allele. The mother and brother do not present any PTLS neurologic/behavioral clinical features. Extensive sequencing of *RAI1* promoter and predicted regulatory regions showed no potential causative variants accounting for gene overexpression. However, the mother and both children share a novel private missense variant in *RAI1* exon 3, currently classified as a VUS (uncertain significance), though predicted by two bioinformatic tools to disrupt the binding site of one specific transcription factor. The reported familial case, the second showing *RAI1* overexpression in the absence of *RAI1* duplication, may help to understand the regulation of *RAI1* dosage sensitivity although its phenotypic effect remains to be determined.

## Introduction

The chromosomal band 17p11.2 is a region prone to rearrangements due to enrichment of highly homologous low-copy repeats [1, 2] and is involved in two different genomic disorders. Deletion and reciprocal duplication within this region are causative of the Smith-Magenis syndrome (SMS, OMIM#182290) [3] and the Potocki-Lupski syndrome (PTLS, OMIM#610883) [1, 4], respectively. The dosage-sensitive *retinoic acid-induced gene 1* (*RAI1*, OMIM*607642), mapping at 17p11.2, is the driver gene of SMS and PTLS phenotypes [5, 6]. About 90% of molecularly diagnosed SMS patients have a recurrent 3.7 Mb interstitial deletion [7] or a non-recurrent larger or smaller deletion, whereas the remainder harbor loss of function mutations or deletions in the *RAI1* coding region [8]. Around 67% of PTLS patients harbor the recurrent 3.7 Mb microduplication and the remainder ones carry either larger or smaller non-recurrent duplications, involving the entire *RAI1* gene [6, 9] leading to its over-dosage. SMS deletions and PTLS duplications are de novo; however, a few familial cases of inherited SMS and PTLS are reported, showing a wide clinical expressivity [10–16]. Some SMS cases of maternal mosaicism are known [10, 11]. Moreover, Acquaviva et al. in 2017 reported for the first time an SMS patient, harboring a *RAI1* frameshift mutation, generating offspring with the same alteration [12]. To date, four PTLS families with the common recurrent duplication have been reported [13, 14, 16]. Of note, in all families the 17p11.2 duplication was maternally transmitted and in a recent paper Grama et al. described a mother and her five children, with a less severe form of the disease, confirming a wide clinical expressivity [16].

RAI1 is a transcription factor, which works as chromatin reader in a multiprotein complex [17, 18], and positively regulates the expression of genes involved in the development and function of the mammalian brain, particularly safeguarding homeostasis of synaptic plasticity [19] and maintenance of circadian rhythm [17, 18, 20].

SMS is a neurodevelopmental disorder with an incidence of 1:15,000–25,000 live births [21], characterized by distinctive craniofacial dysmorphisms, neurological abnormalities including variable intellectual disability (ID), behavioral difficulties (hyperactivity, self-injury, aggression, autistic traits), sleep disturbances, speech and motor delay, and multiple congenital defects [22]. Sleep anomalies, observed in 75–100% of cases, are a hallmark of the syndrome; SMS patients present an inverted rhythm of melatonin secretion and experience difficulties in falling asleep at night, early waking, frequent night-time arousals, and daytime napping [23, 24].

PTLS has an incidence of about 1 in 20,000 live births and shows a phenotype less severe than SMS [25]. PTLS clinical features include speech and language impairment, ID, behavioral problems (attention deficit, hyperactivity, anxiety, autistic traits), infantile hypotonia, failure to thrive, short stature, congenital cardiovascular abnormalities, and mild dyssomnia characterized by difficulties in sleep maintenance [26–29].

In the current study, we report on a 21-year-old girl with a clinical suspicion of SMS who was found to carry a de novo heterozygous intragenic *RAI1* deletion unexpectedly coupled to *RAI1* overexpression, which was also present in her mother and brother.

The allele-specific overexpression involves the proband’s deleted allele confirming SMS clinical diagnosis, while it does not associate with PTLS phenotype in her mother and brother.

## Materials and methods

### Array-CGH

High-resolution array-based Comparative Genomic Hybridization (array-CGH) analysis was performed on genomic blood DNA of the patient, brother and mother, using the SurePrint G3 Human CGH Microarray Kit 2x400K in accordance with the manufacturer’s instructions (Agilent Technologies, Palo Alto, CA, USA). Copy number variants (CNVs) were analyzed and mapped using the Human Genome assembly GRCh37/hg19. CNV classification was performed according to the Database of Genomic Variants (http://projects.tcag.ca/variation/, release March 2016) to exclude common polymorphic CNVs with a frequency >1% in healthy controls.

### Next generation sequencing

Genomic DNA was extracted from whole blood using the GenElute Blood Genomic DNA kit (Sigma-Aldrich, St. Louis, MO, USA). A next generation diagnostic panel, including *RAI1* (NM_030665) and other genes known for their association with SMS, such as *MBD5* (NM_018328) and *HDAC4* (NM_006037), was interrogated. Genomic sequencing of the whole coding region including 20 nucleotides of flanking intron-exon junctions was performed by Illumina Nextera Rapid Capture Enrichment protocol, following the manufacturer’s instructions, while the uncovered genomic regions were analyzed by Nextera-XT-Library-prep protocol (Illumina, San Diego, CA, USA) and Sanger sequencing using the Big Dye Terminator v.3.1 Cycle Sequencing Kit (Thermo Fisher Scientific, Waltham, MA, USA). The clinical effect of variants was assessed using the InterVar classify system tool (http://wintervar.wglab.org) [30], based on the officially published ACMG guidelines [31], and focusing on the inheritance.

### MLPA

Multiplex Ligation-dependent Probe Amplification assay (SALSA MLPA probe mix P369-A2, MRC-Holland, Amsterdam, The Netherlands) was performed in accordance with the manufacturer’s instructions. MLPA probes map at chr17:17585042-17585096 (exon 1), chr17:17585180-17585237 (intron 1), chr17:17627319-17627382 (exon 2), chr17:17627646-17627720 (intron 2), chr17:17696251-17696314 (exon 3), chr17:17706996-17707092 (exon 4), chr17:17712712-17712784 (exon 5), and chr17:17714172-17714243 (exon 6) (GRCh37/hg19 assembly).

### Amplification of the deletion junction fragment

To localize the deletion breakpoints, long-range PCR was performed on genomic DNA using TaKaRa LA Taq™ kit (TaKaRa, JP) and on cDNA using the KAPA2G Robust PCR Kit (KAPA BIOSYSTEMS, Wilmington, MA, USA) (Primers details are reported in Supplementary Table S1). Amplicons were sequenced using the Big Dye® Terminator v.3.1 Cycle Sequencing kit (Thermo Fisher Scientific). Deletion junction sequences were aligned to the human reference genome sequence (human genome assembly GRCh37/hg19), and electropherograms analyzed with the ChromasPro 1.5 software (Technelysium Pty Ltd., Tewantin QLD, Australia).

### RT-qPCR

For quantitative gene-expression analysis, total RNA of patient, her brother, parents, and ten healthy controls, was collected using Tempus Blood RNA tubes (Thermo Fisher Scientific), isolated using the Tempus Spin RNA Isolation kit (Thermo Fisher Scientific), and reverse-transcribed using the High-Capacity cDNA Reverse Transcription kit (Thermo Fisher Scientific). RT-qPCR was performed using a QuantStudio 12K Flex Real-Time PCR System (Thermo Fisher Scientific). The amounts of mRNA were calculated using the 2^−ΔΔCt^ method, normalized against housekeeping genes *GAPDH* and *TBP*. RT-qPCR reactions were carried out using the TaqMan method (TaqMan ID# Hs00430773_m1 *RAI1* NM_030665 ex 2-3, Hs01554690_m1 *RAI1* NM_030665 ex 3-4, Hs99999905_m1 *GAPDH*; Hs00427620_m1 *TBP*), and SYBR Green methodology (Primers details are reported in Supplementary Table S2). Data were analyzed using the QuantStudio 12K Flex Software v1.2.3 (Thermo Fisher Scientific). We established the proper range of gene expression in ten healthy controls by calculating the mean value ±2 standard deviation.

### Sequencing of *RAI1* noncoding regions

Promoter and regulatory regions selected based on the presence of predicted elements by UCSC browser (Tracks: Integrated Regulation from ENCODE → Layered H3K4Me1; ENCODE Histone Modification → Broad ChromHMM) [32], were amplified using the AmpliTaq Gold DNA Polymerase Kit (Thermo Fisher Scientific) or, in the case of CG-rich regions, the KAPA2G Robust PCR Kit (KAPA BIOSYSTEMS). A full list of primers pairs, and detailed conditions are reported in Supplementary Table S3.

### Transcription factor binding prediction

PROMO (http://alggen.lsi.upc.es/cgi-bin/promo_v3/promo/promoinit.cgi?dirDB=TF_8.3) [33, 34], and JASPAR (http://jaspar.genereg.net/) tools were used to analyze putative transcription factors binding sites.

## Results

### Clinical report

**Fig. 1:**
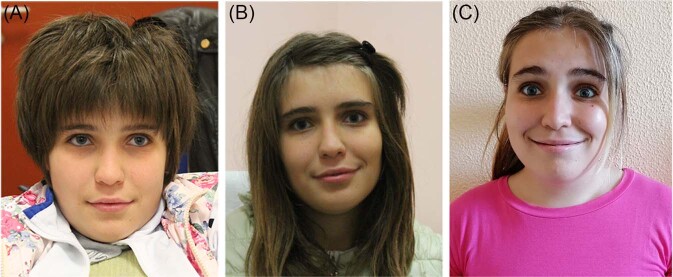
Facial features of the patient. Front facial view of the patient at 15 (**A**) 17 (**B**) and 21 years (**C**), showing brachycephaly, broad face, midface hypoplasia, and thick eyebrows.

The proband, currently 21 years old, was referred to our lab for suspected diagnosis of SMS. She is the first child of unrelated healthy parents, born at term by cesarean section (podalic presentation). The mother and father have an uneventful family history for ID or any other relevant genetic conditions and at time of birth were 28 and 31 years old, respectively. The proband’s birth weight was 3.1 kg (50th centile), while length and occipital frontal circumference are not known. Apgar scores were 9/10. The girl was referred to our lab when she was 15 years old. Clinical examination revealed mild craniofacial dysmorphism slightly resembling SMS: in detail she showed brachycephaly, midface hypoplasia, broad face, and thick eyebrows (Fig. 1), brachydactyly, mild overweight (64.5 kg versus 160 cm height and 24.9 kg/m^2^ BMI), and hoarse voice. By using Face2Gene (https://www.face2gene.com/) the patient was not associated to SMS with a high/medium score.

Moreover, she presented hypotonia, developmental delay, mild language impairment, sleep disturbance, and neurobehavioral manifestations such as hyperactivity, self-destructive and aggressive behavior, attention deficit, limited social interactions, hyperphagia, and stereotypies. Brain MRI showed no structural defects, while EEG revealed widespread epileptiform abnormalities in asleep and after awakening: however, she never had seizures. Due to delayed developmental milestones neurocognitive assessment was performed with WESCHLER scale (WISC-IV) at the age of 11 indicating a moderate ID (total IQ 44) with a homogeneous profile between verbal (IQ 54) and performance skills (IQ 46). She attended high school until age 16 with individual support and full-time assistance. From the second to the last evaluation, at age 18, her speech delay was characterized by greater involvement of expressive language, simplification in sentence construction, and semantic and pragmatic limitations of content. Moreover, a worsening of behavioral problems was observed, with persistent aggressiveness and compulsively putting hands and objects into the mouth. To ameliorate the neurobehavioral problems, several drugs were administered (Risperidone, Aripiprazole, Valproic Acid) without benefit. As regards sleep disturbance, characterized by waking up at around 3 in the morning with subsequent difficulty falling asleep, this persisted despite a moderate improvement after treatment with Chlorpromazine. At the last evaluation (at age 21), behavioral problems remain more significant than speech delay.

### Characterization of proband’s *RAI1* intragenic deletion

High-resolution array-CGH analysis did not reveal any 17p11.2 deletion and CNVs of clinical relevance in the patient and in her family. Targeted NGS mutational screening did not identify any pathogenic de novo variants, apart from a *RAI1* heterozygous missense unreported variant, c.3272C > A, inherited from the healthy mother and also transmitted to the brother (Supplementary Fig. S1A), and currently classified as VUS according to the Varsome software [35].

**Fig. 2:**
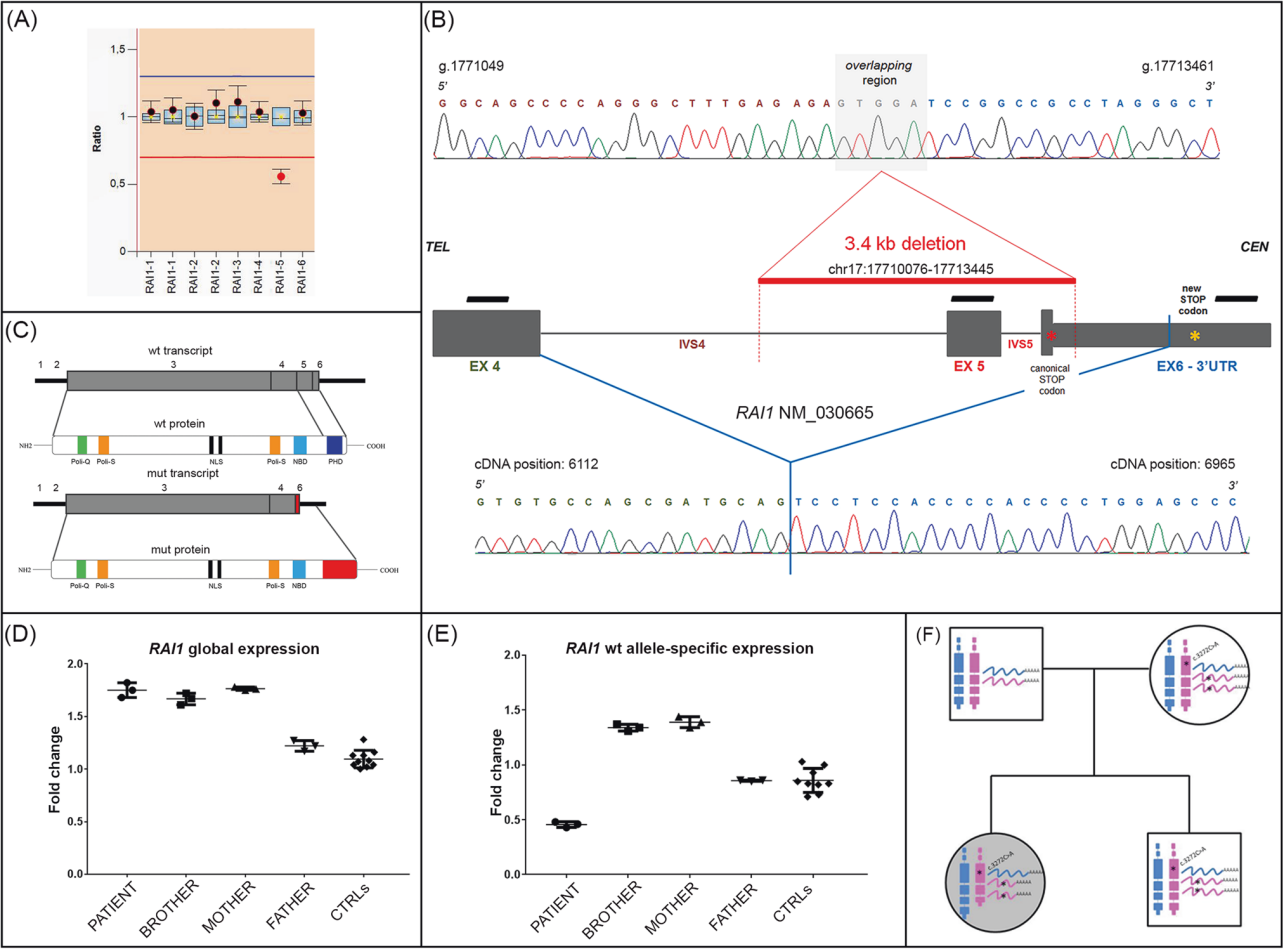
Molecular characterization of *RAI1* intragenic deletion and expression analyses. **A** MLPA profile’s probes for *RAI1* gene reveal in the proband a heterozygous deletion of *RAI1* involving exon 5. Each black dot displays the final probe ratio for each locus analyzed in the patient compared to controls. Standard deviations were set up according to the Coffalyzer DB software v131211. **B** Graphic representation of *RAI1* deletion characterized in the patient at genomic and transcript levels. Top panel: electropherogram showing the deletion breakpoints at genomic level. The 3.4 kb *RAI1* deletion is represented by a red bar involving part of IVS4, exon 5, IVS5 and part of exon 6 (chr17:17710076-17713445, hg19). Black bars above *RAI1* exons refer to MLPA probes. Bottom panel: electropherogram of the aberrant *RAI1* transcript, derived from the activation of a new splice-site in exon 6. **C** Wild-type and mutated *RAI1* mRNAs and corresponding proteins with the insertion of 64 new amino acids highlighted in red. **D** Scatter plots obtained using TaqMan probe on exon junction 3-4, showing global *RAI1* expression in patient (circles), brother (squares), mother (triangles pointing upward), father (triangles pointing downward) relatively to normal controls (rhombuses). The horizontal black bars indicate the range between mean ±2 standard deviation values. Data were normalized against *TBP* housekeeping gene. Similar results were obtained after normalization against *GAPDH* housekeeping gene (data not shown) and by using the Taqman probe on exon junction 2–3 (data not shown). **E** Scatter plots using the Syber green probe designed across exon junction 4–5, involved in the deletion showing specific expression of wt *RAI1* transcript in:patient (circles), brother (squares) and mother (triangles pointing upward), respectively. Data were normalized against *TBP*. Similar results were obtained after normalization against *GAPDH* housekeeping gene (data not shown). **F** Family tree summarizing the *RAI1* genotype and transcripts.

MLPA analysis revealed in the patient a pathogenic de novo heterozygous deletion encompassing *RAI1* exon 5 (Fig. 2A). Long-Range PCR and amplicon sequencing finely characterized the deletion breakpoints at nucleotide level, establishing, in addition to exon 5, the partial involvement of exon 6. In detail, the deletion has a total length of 3.4 kb, starting within IVS4 (2633 bp upstream exon 5) and ending within exon 6 (144 bp downstream the stop codon) (Fig. 2B). A run of 5 bp (GTGGA) microhomology was found both in IVS4 and exon 6; considering the repeated GTGGA sequence as part of IVS4, the deletion was mapped at chr17:17,710,076-17,713,445 (hg19). cDNA sequencing of the deleted allele revealed that a larger portion of exon 6 was missing. This finding could be attributed to the loss of the canonical acceptor splice site and the creation of a new one downstream from the genomic breakpoint (Fig. 2B). Moreover, the deletion causes the loss of the canonical stop codon which is replaced by a new one located 946 bp downstream. Due to the deletion, a protein lacking the last 20 amino acids encoding for the PHD functional domain, and presenting the insertion of 64 new amino acids is predicted (Fig. 2C).

### Quantitative expression analysis of proband’s deleted allele

Next, RT-qPCR studies were carried out using TaqMan assay for *RAI1* exons 2–3 and 3–4 junctions, showing an unexpected and significant increase in transcript levels in the patient’s, brother’s and mother’s peripheral blood cells compared to the father’s and ten healthy controls (Fig. 2D).

To evaluate a possible allele-specific expression, we performed RT-qPCR experiments, based on SYBR Green methodology, using specific primers for the exon junction 4–5 designed to specifically amplify the non-deleted allele of the patient. The analysis showed in the proband an expression of the transcript which was half that of the controls and one-third compared to mother and brother (Fig. 2E). By comparing these data with the previous results of RT-qPCR we conclude that the overexpression is allele-specific in the patient, brother and mother, and involves the proband’s deleted allele, inherited from the mother (Fig. 2F).

### Search of variants shared by family members with the overexpressed *RAI1* allele

With the aim of identifying a putative in cis variant, shared by mother and both children and accounting for allelic-specific *RAI1* overexpression, the gene promoter and predicted regulatory regions (43,921 bp, Supplementary Fig. 2), were sequenced but no variants were identified. ENCODE data (chromatin state segmentation and H3K4me1 histone mark) [31] were used to examine the *RAI1* transcriptional regulatory elements including promoter, enhancer, insulator, CpGs island regions and the antisense transcript RAI1-AS1. Thus the only candidate variant shared by the three family members remains the missense variant, c.3272C > A, located in exon 3, which the PROMO tool predicts to cause the lack of the binding site of three different transcription factors: E2F-1, GR-alpha (encoded by *NR3C1*) and AP-alpha A (encoded by *TFAP2A*) (Supplementary Fig. S1B). This prediction is supported, at least for AP-alpha A, by the JASPAR database.

## Discussion

SMS and PTLS are two rare and distinct neurodevelopmental disorders caused by deletion and duplication of the dosage-sensitive gene *RAI1*, respectively. The two syndromes cannot be strictly considered “mirror syndromes” as they appear to share some aspects (i.e., hyperactivity and anxiety), but are very dissimilar for others (i.e., large variability and different gestalt observed in PTLS) [26, 36]. However, in general, PTLS features are milder than those observed in SMS, supporting the notion that genomic duplications are generally better tolerated than the corresponding deletions.

We present a patient characterized by mild craniofacial distinctive SMS dysmorphism (Fig. 1) and a typical SMS neurological and neurobehavioral phenotype, who carries a pathogenic 3.4 kb de novo deletion encompassing the whole of *RAI1* exon 5 and portion of exon 6. Despite Face2Gene not supporting the clinical hypothesis (“low gestalt”) our clinician (DM) highlighted the similarity of the ocular region with SMS. This observation was retrospectively evaluated an earlier photo of the girl, which increased the system score to medium, consistent with the clinical hypothesis. The unique intragenic deletion of the proband could explain the lack of a full SMS phenotype, thus impairing the comparison with the “classic” SMS patients.

Intragenic *RAI1* rearrangements are very rare, and to date in literature only another two *RAI1* intragenic deletions have been reported in SMS patients, showing different breakpoints and locations: a 140 kb deletion involving exons 1 and 2 [37], and a 29 bp deletion located in exon 3 [8].

Interestingly, a concomitant *RAI1* overexpression has been identified, in our patient and her brother and mother, by allele-specific quantitative expression analysis. The proband inherited the maternal overexpressed *RAI1* allele, target of a meiotic/mitotic error leading to *RAI1* intragenic deletion, consistent with SMS clinical diagnosis. As the overexpressed allele is coincidentally the deleted one, the mechanism of dosage compensation by an overexpressed *RAI1* wt allele which might potentially account for the relatively mild proband’s phenotype, could be ruled out. Another genetic mark exclusive to our patient is the atypical intragenic deletion which, unless the altered transcript is degraded by nonsense medicated decay, is predicted to code for a truncated protein lacking the PHD domain (Fig. 2C), involved in the chromatin interactions as histone code reader. Should a truncated protein be present, it might exert some residual activity influencing the proband’s mild SMS phenotype.

As the overexpression of the maternal allele was indirectly demonstrated, we speculate that the patient, brother and mother may harbor a variant *in cis* within the *RAI1* overexpressed allele that can favor its upregulation. Sequencing of the regions containing the predicted regulatory elements of *RAI1* did not identify any variant shared between family members with *RAI1* overexpression. However, as not all *RAI1* noncoding regions have been sequenced we cannot exclude the presence of other variants that run with the *RAI1* overexpression and further analyses are needed.

Currently, the only shared heterozygous variant found is the yet-unreported missense variant, c.3272C > A, located in exon 3. Interestingly, in the presence of this C > A sequence change prediction tools indicate a lack of the binding site of the AP-alpha A transcription factor. The AP-alpha A, binds the consensus sequence 5′-GCCNNNGGG-3′ and activates the transcription of some genes while repressing or decreasing the transcription of others [38]. So we hypothesize that the shared c.3272C > A variant might interfere with the interaction of AP-alpha A acting as a repressor. Functional studies, aimed at clarifying the effect of this rare variant on *RAI1* expression, need to be performed to demonstrate this enticing hypothesis.

PTLS is generally due to 17p11.2 duplications, causing *RAI1* overexpression. One single PTLS case has been reported without 17p11.2 duplication but bearing a maternal inherited deletion located upstream from the *RAI1* promoter [15]. In this familial case both patient and mother showed *RAI1* overexpression in lymphoblast cell lines. It is worth noting that the mother was reported to manifest learning difficulties and significant sensory issues [15], confirming PTLS high phenotype variability. In our familiar case, according to phenotype evaluation, the patient’s brother and mother do not show any neurological/behavioral clinical signs matching the PTLS phenotype. The clinical re-evaluation of the mother revealed the presence of some facial characteristics, namely triangular face, broad forehead, smooth philtrum, micrognathia, long nasal tip, and thin upper lip, mimicking the facial appearance of the syndrome (Supplementary Fig. S3). However, the lack of other physical and neurobehavioral features did not lead to a PTLS diagnosis, which has never been suspected for her son. The lack of PTLS clinical diagnosis may be accounted for by the broad phenotype variability of the syndrome as previously reported [13–16]. However, we cannot exclude that *RAI1* overexpression identified in blood might be tissue-specific and therefore not present in other tissues, particularly the nervous system, a scenario which might also explain the lack of a PTLS phenotype in the patient’s brother and mother. The generation of patient-specific induced pluripotent stem cell-derived neurons might allow the verification of the tissue-specific hypothesis.

In conclusion, gene expression analysis combined with standard genetic analysis can provide additional insights into the diagnostic flow chart of SMS by detecting changes in *RAI1* dosage in cases with a robust clinical diagnosis but no identified mutation in the *RAI1* coding/splicing region. We have described the second report of *RAI1* overexpression in the absence of *RAI1* duplication. The overexpression is found in the proband’s brother and mother who do not present PTLS features, and intriguingly involves the proband’s *RAI1* allele which harbors a de novo *RAI1* intragenic deletion. Disruption of *RAI1* genomic structure corroborates the proband SMS phenotype and accounts for her distinctive mild phenotype with the exception of self-destructive and aggressive behavior.

## Supplementary information

Supplementary Informations clean file

## Data Availability

The data underlying this article are available in the article. The *RAI1* intragenic deletion and the missense variant, c.3272C > A are publicly available at ClinVar database (http://www.ncbi.nlm.nih.gov/clinvar/): accession numbers SCV002014620 to SCV002014621.
