# Supplementary material for: A unique Smith-Magenis patient with a de novo intragenic deletion on the maternally inherited overexpressed RAI1 allele
Source: Eur J Hum Genet. 2022 Jul 11;30(11):1233–8. doi: 10.1038/s41431-022-01143-5 (PMC9626456; doi:10.1038/s41431-022-01143-5)
Supplement: Supplementary file 1 — Supplementary Informations clean file [file 41431_2022_1143_MOESM1_ESM.docx]

**Supplementary Figures**

**
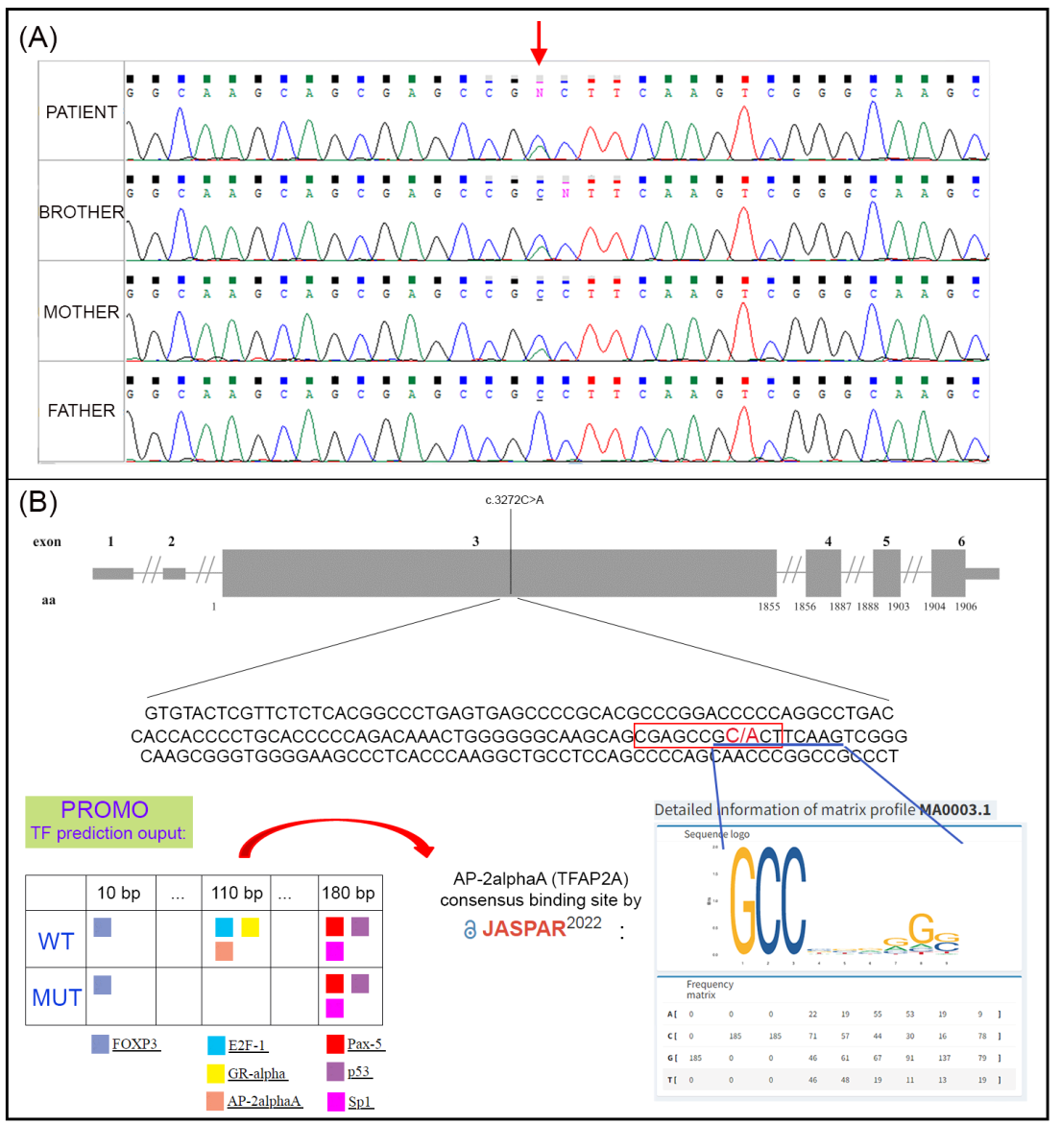
**

**Fig. S1.** (**A**) Sanger sequencing confirmation of the missense variant, c.3272C>A (red arrow) shared by patient and her brother and mother. (**B**) *In silico* prediction by PROMO tool (version 3.0.2), shows that the variant, c.3272C>A (in red within the red-framed rectangle), leads to the loss of the binding site of three transcription factors, E2F-1, GR-alpha and AP-alpha A. Bottom left: the colored small squares identify the specific transcription factors. Bottom right: according to the Jaspar database (9^th^ release, 2022), the consensus sequence with high frequency matrix (matrix profile MA0003.1) of transcription factor AP-alpha A (TFAP2A) well matches the region where c.3272C>A maps (blue line).


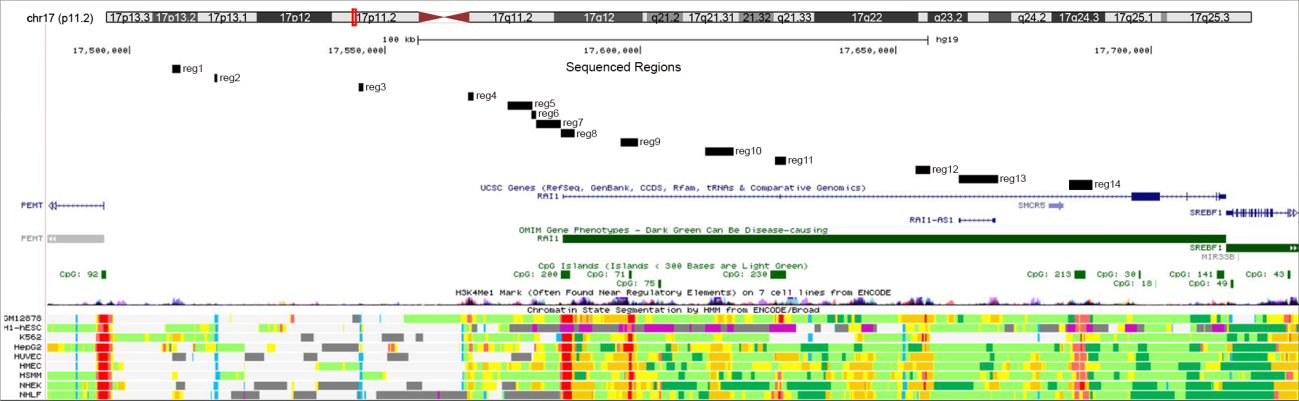


**Fig. S2.** UCSC genome browser view of 17p11.2 region where *RAI1* maps (chr17:17483937-17728959, hg19). UCSC genes are depicted in blue, and OMIM genes in green. Regulatory elements predicted by ENCODE on the basis of chromatin state segmentation in nine human cell lines are depicted in different colors: red, active promoter; yellow, weak enhancer; orange, strong enhancer; dark green, transcriptional elongation; light green, weak transcribed; light blue, insulator; dark grey, polycomb-repressed; light grey, heterochromatin. Violet/light-blue spikes represent methylation of H3K4 sites., The black bars at the top represent the selected regions sequenced (reg1, chr17:17508446-17510048; reg2, chr17:17516672-17517129; reg3, chr17:17544898-17545881; reg4, chr17:17566260-17567385; reg5, chr17:17574032-17578970; reg6, chr17:17578818-17579683; reg7, chr17:17579657-17584467; reg8, chr17:17584419-17587127; reg9, chr17:17596278-17599551; reg10, chr17:17612671-17618254; reg11, chr17:17626371-17628581; reg12, chr17:17653916-17656865; reg13, chr17:17662327-17670132 (RAI1-AS1); chr17:17683944-17688543, reg14).


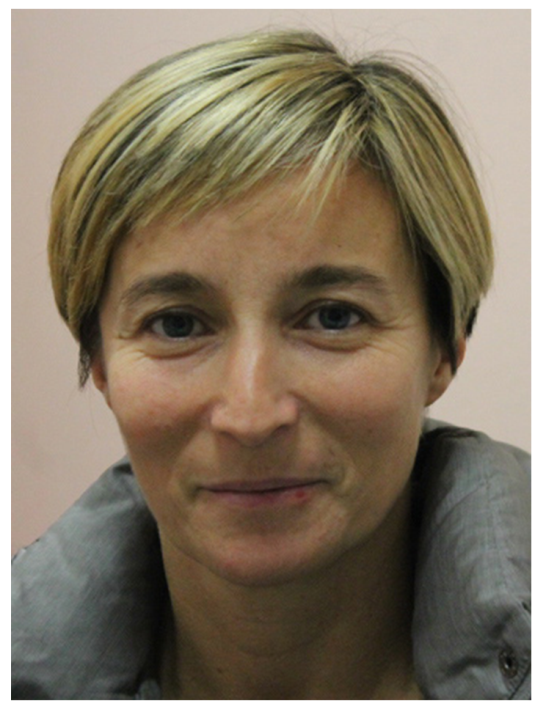


**Fig. S3.** Facial features of patient’s mother at 44 years: triangular face, broad forehead, smooth philtrum, micrognathia, long nasal tip, thin upper lip can be observed.

**Supplementary Tables**

**Table S1. Primers used for amplification of the deletion junction fragment**

|  | Primer sequence (5'->3') | Ta (°C) | PCR size (bp) |
| --- | --- | --- | --- |
| LR-PCR on gDNA | Fw: TAGCAACTGTTTAGTGGTGGGACTTGTA  chr17:17,709,560-17,709,587, hg19 | TD:69.1🡪62.1 | 4771 |
|  | Rev: CAAAGGCCCGGTTGGGGCTCTGTT  chr17:17,714,307-17,714,330, hg19 |  |  |
| LR-PCR on cDNA | FW: AAAGGATGCCTCCACACCTACCACTAC  chr17:17,707,118-17,707,144, hg19 | 66.6 °C | 1093 |
|  | Rev: CAAAGGCCCGGTTGGGGCTCTGTT  chr17:17,714,307-17,714,330, hg19 |  |  |

**Table S2. Primers used in RT-qPCR analysis of wt *RAI1* transcript**

|  | Primer sequence (5'->3') | PCR size (bp) |
| --- | --- | --- |
| *RAI1* | Fw: ACCTACCACTACCCGTGTGC (ex4)  chr17:17,707,133-17,707,152, hg19 | 77 |
|  | Rev: TGTTTGGGACATTTCAAAGAA (ex5)  chr17:17,712,729-17,712,749, hg19 |  |
| *GAPDH* | Fw: GGGAAGCTTGTCATCAATGGA (ex4)  chr12:6,645,913-6,645,933, hg19 | 73 |
|  | Rev: CGCCCCACTTGATTTTGG (ex5)  chr12:6,646,097-6,646,114, hg19 |  |
| *TBP* | Fw: GTTCTGGGAAAATGGTGTGC (ex5)  chr6:170,876,064-170,876,083, hg19 | 100 |
|  | Rev: GCTGGAAAACCCAACTTCTG (ex6)  chr6:170,878,746-170,878,765 |  |

*

**Table S3. Primers targeting *RAI1* noncoding regions with predicted regulatory elements**

|  | Primer sequence (5'->3') | Ta (°C) | PCR size (bp) |
| --- | --- | --- | --- |
| **Reg1** |  |  |  |
| Reg1_a | Fw:CAAGTGCCTCACAGGGAGT Rev:CGTGGTGTGTACCTACAGTGG | 60.3 | 585 |
| Reg1_b | Fw:GCTCAGATCCTTTGCCATTT Rev:GGGGAAGGAGTAGGAGGAGA | TD:61.2🡪54.2 | 565 |
| **Reg2** |  |  |  |
| Reg2 | Fw:ACTCAAAGCCAGGTCCCTCT Rev:AATTCAGTGCGGTCTCCATC | 58.3 | 458 |
| **Reg3** |  |  |  |
| Reg3_a | Fw:CCACAGTTCCTACACCACTGA Rev:CCAAGGGCACATGGTAATTCC | 59.6 | 458 |
| Reg3_b | Fw:GTTCAATTCTGCCCCAACAC Rev:TTGATGAGCTCTTCGTGGAAG | 57.5 | 400 |
| Reg3_c | Fw:TATCCTGCCAAGAGATCATCC Rev:TCGGATACAGCAAAATGAAGC | 56.7 | 350 |
| **Reg4** |  |  |  |
| Reg4_a | Fw:TCAGCGGAGGAAGTCTCTGT Rev:GGGCTGTCAGTCATCAAGGT | 59.3 | 538 |
| Reg4_b | Fw:CCATCCCCCTACACAAACAC Rev:CAGAACCAACTCTGGTGCAA | 58.3 | 640 |
| **Reg5** |  |  |  |
| Reg5_a | Fw:CTGTGCCACTGCTACCCTCT Rev:GCGTGTGTGTGTGCTTCCTG | 61.3 | 434 |
| Reg5_b | Fw:TGAGGATGGAAGCAAGGATT Rev:CGAATTTCTTGAAGGCAGGA | 55.2 | 355 |
| Reg5_c | Fw:TTGGGAAGCATAAGCGAGTT Rev:CCAAGCAATTGTTCCTTTCC | 55.2 | 488 |
| Reg5_d | Fw:TTGAGCTAAGCCTGGAAGGA Rev:TCACTGTCATTGCCATCTCG | 57.2 | 430 |
| Reg5_e | Fw:GAAGAAGGTGGGTGAGCAGA Rev:TGCTTTCAGGCCCTACAGAT | 58.3 | 438 |
| Reg5_f | Fw:AGGTGAGGAAATGGCTTTCA Rev:CCTCCAACCAGCTTTCATGT | 56.2 | 442 |
| Reg5_g | Fw:GACAGTGGTGAATGCAGGAG Rev:GGAATGGGGAGAAAAGGAGA | 58.3 | 428 |
| Reg5_h | Fw:CGGCCCTTTAGATCTTTGAA Rev:AGGATGCAGAGATTGTCACG | 56.2 | 430 |
| Reg5_i | Fw:CGAACCATTCCTGAACCAAT Rev:GGGGCACCCTAATCATACAA | 56.2 | 319 |
| Reg5_j | Fw:CCGGCTTAAACAAGATGGAA Rev:CACGTTGGGGGTTTTCATTA | 55.2 | 424 |
| Reg5_k | Fw:GTGGCTGACAAACCAAGGAT Rev:TCTTCAGAAGCATGAGCCAAA | 56.5 | 349 |
| Reg5_l | Fw:AGGCAATTTTGTGGGAAGAA Rev:ATGCCCGGCTGATTTATGAT | 54.2 | 429 |
| Reg5_m | Fw:AGGGTTTTGCCCTTATGACC Rev:TTGACTGTCCCCTCCAAAAC | 57.2 | 430 |
| Reg5_n | Fw:AGCCTGGGTGACAAGAACAA Rev:CCGAGCCAAACCTCATTCTA | 57.2 | 418 |
| Reg5_o | Fw:CCCTTCTTTAGAGTGGGTTCAA Rev:CATCAGCCTCCAGCACTTCT | 58.7 | 684 |
| **Reg6** |  |  |  |
| Reg6_a | Fw:CTCTGGGCACTCACAAGACC Rev:GTGCAGGAGGACTCCCATTA | 60.3 | 471 |
| Reg6_b | Fw:AGGCCTCTATGTTCTCTGCAA Rev:TTGGGTTTCAGACACCTTCC | 57.5 | 583 |
| Reg6_c | Fw:CCAGGCAGGAAACATGATCT Rev:GAGGATCACTTGAGCCCAGA | 58.3 | 585 |
| **Reg7** |  |  |  |
| Reg7_a | Fw:CCAGGCAGGAAACATGATCT Rev:AAATCACCTGGCTGACCAAC | 57.2 | 198 |
| Reg7_b | Fw:CAAGGCCACACAGATGTGAGT Rev:TGGGGCAGAGTCTCAAACTT | 58.4 | 693 |
| Reg7_c | Fw:TGCTCTGCTTTCCAATCAAA Rev:TGATTTTCCTCTGCCTGCTT | 54.2 | 366 |
| Reg7_d | Fw:GACTGAGCCCTCTCAACCAG Rev:GGACCCTCTGAATGCTGTCT | 60.3 | 418 |
| Reg7_e | Fw:CACCACAGCACTCTTCATGG Rev:TGCCTGTGTTTCTCAGCTTG | 58.3 | 417 |
| Reg7_f | Fw:AGGAAGGTCAAGGGAGGAAG Rev:CACATACCACACCACCAAGC | 59.3 | 326 |
| Reg7_g | Fw:CATGGTGAAACCCACTCTCC Rev:CAGGTGCCTAGGGATGTTGT | 59.3 | 287 |
| Reg7_h | Fw:CCGAGAGGCAGAGGTTACAG Rev:AGGAATATGCAGGCAGATGG | TD:63.3🡪56.3 | 574 |
| Reg7_i | Fw:GCAGTTGTTAGTACCTTGACC Rev:CACAAGGGTCCCCAAGACTA | 58.5 | 432 |
| Reg7_j | Fw:TCCAAGGCCAGAAAATTGAC Rev:TCATGGGTCTGTTGAGAGGA | 56.2 | 365 |
| Reg7_k | Fw:TCTAGCATCTTAAAGCAGTAATA Rev:AAGTGCGGTTTCTGGATGAC | TD:59.2🡪52.2 | 559 |
| Reg7_l | Fw:GGCAAAACTCCGTCTCAAAA Rev:AGCCTCCCTCATTTGTCCTT | 56.2 | 550 |
| Reg7_m | Fw:TTTGCAGATGCAGGATTCAC Rev:TGGGCACATTGAAGTCAGAG | 56.2 | 403 |
| Reg7_n | Fw:GTGGCTACCTTCCTTGCACT Rev:GAGAAGGAGGCCTGTCTGTG | 60.3 | 409 |
| Reg7_o | Fw:TGAGGCCAGGAATTCAAGTT Rev:CACCATTAGCCCTCACCTGT | TD:61.3🡪54.3 | 429 |
| **Reg8** |  |  |  |
| Reg8_a | Fw:ACAGGTGAGGGCTAATGGTG Rev:ACTCGCTCTCCCACTCGTCT | 60.3 | 464 |
| Reg8_b | Fw:GCTCGTCCGCTCTTCCTG Rev:CACACCACACAAAGCAAGGA | TD:63🡪56 | 539 |
| Reg8_c | Fw:ATCCTAGGCCGGGTGATG Rev:GTCACGCATGGGGAAGTC | 58.4 | 384 |
| Reg8_d | Fw:GAGTGTGGCAAGGGATCTG Rev:GCTACAGAGCTCCCCAGGT | 60 | 327 |
| Reg8_e | Fw:CTCTCTGCGGTGTCCCTACC Rev:CAAGAGCCCCAAGAAAGAAA | TD:61.2🡪54.2 | 453 |
| Reg8_f | Fw:GAGGGGAAGCGAAACACC Rev:TGCCCTTGAATTCTCAACAC | TD:60.8🡪53.8 | 448 |
| Reg8_g | Fw:ACGGTTCCTCCCACCAATAC Rev:TGAGCAGAGCGAGGACTGTA | 59.3 | 333 |
| Reg8_h | Fw:TGGGTTAGGCTAGCTCTGATG Rev:TCCTATTTTGCCCACTCCAC | 58.4 | 491 |
| **Reg9** |  |  |  |
| Reg9_a | Fw:GAGAAGGTGGTGTGGAATGC Rev:TCTCCCCTCTCCTTTTGTGA | 58.3 | 417 |
| Reg9_b | Fw:TCATTCCTCCACCTCCTCTG Rev:ACAAAGCCCTGTTTGTTTGC | 61.3 --> 54.3 | 470 |
| Reg9_c | Fw:CGGCAGGTAGCTGAGAAAGA Rev:CAATGACGGGACAAGGGTAG | 59.3 | 413 |
| Reg9_d | Fw:ACGTAGGACTCGCTGGCTTA Rev:CGTCTTCCTGGCTATGTTCC | 59.3 | 365 |
| Reg9_e | Fw:AGGCCTGAGGAACCAAACTG Rev:GGCCTGACAGCTCCTACCTA | 60.3 | 229 |
| Reg9_f | Fw:ACCTGGCCCACCTCTCAG Rev:CAACGCTGGGACTGGAAG | 59.6 | 476 |
| Reg9_g | Fw:GGTCTTTTTCCGGGAACG Rev:CCCTTCTTTCTCCTGCCC | 57.3 | 379 |
| Reg9_h | Fw:GCCTCTCAGCTGCAGTCTCT Rev:AGGCGGCTGTGGAGTAGTAA | 60.3 | 355 |
| **Reg10** |  |  |  |
| Reg10_a | Fw:ACTCCTACCCCGACTCTCTT Rev:CCTCCTGGGTGCTCATTCA | 59.3 | 460 |
| Reg10_b | Fw:GTGCAGCTTCCAGAATCTT Rev:CCGCTGGGTTCTGATTCTCT | 58.3 | 426 |
| Reg10_c | Fw:CACATACACTTGGCACCAGG Rev:GGGAGTCCTAGTCTTGGTGG | 60.3 | 436 |
| Reg10_d | Fw:GTGAGTCAGAGGCTGCCA Rev:CAGGAGAGCTCTGGAGTACG | 59.9 | 412 |
| Reg10_e | Fw:TCTGTAGGTGGGGACCTTTG Rev:AGGGCCTGTGTGTTTCTGTC | 59.3 | 473 |
| Reg10_f | Fw:GCCTGAGAGGCTGGTGATAG Rev:GTCCATCCCGTGTACACCTC | 61.3 | 419 |
| Reg10_g | Fw:GCTTGCCATTCAGTCCTTTC Rev:TGAATGCTTTGTCCCTCCTC | 57.2 | 425 |
| Reg10_h | Fw:CAACGAGAGCAAAACTCCATC Rev:AGGCATGCAAGCAGAACAG | 57.2 | 455 |
| **Reg11** |  |  |  |
| Reg11_a | Fw:CCACACAACTGCCCTCTGGA Rev:GGGGTGTGAGTGTAAGAGTGC | 61.5 | 415 |
| Reg11_b | Fw:CTAAACGCACAGCCAAGACC Rev:GCGCCCTCAGTAGCCATTATT | 59.5 | 465 |
| Reg11_c | Fw CATCGCGCTTACCGAGTGGA Rev:CCCGCCGAAAACACAAAGCTC | 61.5 | 402 |
| Reg11_d | Fw:CTCACATTTGTTTCTCCCAAGG Rev:CTTCCGCCAATACCACATTC | 57.7 | 520 |
| Reg11_e | Fw:CTCCCCTTTCCTCCACAAAG Rev:TCCAGGGGAAGTGTTTGAAGC | 59.5 | 417 |
| Reg11_f | Fw GATGGGAAACTTCCCGAGAG Rev:CTGAAGTCACCAGCCAATCAC | 59.5 | 552 |
| **Reg12** |  |  |  |
| Reg12_a | Fw:TGCACCATTGTACTCCGTCT Rev:GAAGGCGGAAGGAAACAGAT | 57.2 | 328 |
| Reg12_b | Fw:TCTGGTTTTGTGCTTGTCCA Rev:TTGATGAGGCTCCACTTCCT | 56.2 | 346 |
| Reg12_c | Fw:TTGACAAGTGCTGGCATGTT Rev:TACGGACAAGGGGAAAACAC | 56.2 | 414 |
| Reg12_d | Fw:CTTGCCCAGAACAGTGACCT Rev:GGGAACGGGTACTAGGTTCG | 60.3 | 439 |
| Reg12_e | Fw:CTCTTCCTGGAGAGCCACAG Rev:GGGCCTCCACTTCCTAGTCT | 61.3 | 413 |
| Reg12_f | Fw:GATCTCAAGCACCCGAACAT Rev:CGCGCCAGTACAAGACCAG | 63.1 --> 56.1 | 419 |
| Reg12_g | Fw:TTTCCTGCCTTTTGTGTTCG Rev:GAGTGTCCAGGAATGACGTG | 61.3 --> 54.3 | 500 |
| Reg12_h | Fw:GGGCCGACCACCTTACTT Rev:GGTGAATGGCCTACTGTGCT | 58.9 | 494 |
| Reg12_i | Fw:GACTTGGGAGGAAGGGAGAC Rev:GGGTAAGGGGTGTGGAGATT | 60.3 | 385 |
| **Reg13** |  |  |  |
| Reg13_a | Fw:TTTGGGTTTGGGGAGTATCA Rev:TGCTGAATGTTCTGCAATGA | 54.2 | 392 |
| Reg13_b | Fw:AGAGTGGCCAACTACGAGGA Rev:ACCCCGAAATATGTGCAGAC | 58.3 | 380 |
| Reg13_c | Fw:CCCTCAGTGACCTGCTTTTC Rev:CTACAGCCCATCTCCTCCAG | 60.3 | 415 |
| Reg13_d | Fw:TTAAGGGCTCTGGATGCTTG Rev:CTGCTCCCTCCCATAAATCA | 57.2 | 387 |
| Reg13_e | Fw:GCTCGAGCAGTGCTGTAATG Rev:CTCCTTGGTGTGGGACTCTG | 60.3 | 397 |
| Reg13_f | Fw:GAGGGTTTATGGCAGCTCAG Rev:CTATAGGCACGCCTTGATGC | 59.3 | 428 |
| Reg13_g | Fw:GAAAACCCTTCCTCCTGCTT Rev:CACATATGGGTCGCCTCTTT | 57.2 | 420 |
| Reg13_h | Fw:CAGTGGAAATGATAATGATAGACCA Rev:TGATGCCTGCCCTAGATACC | 58.4 | 429 |
| Reg13_i | Fw: AGCTGTGGATCCTGACCTGT Rev:CCTCAGTGACCACCACCTTT | 59.3 | 398 |
| Reg13_j | Fw:GGCAGGGACAGTGTCTGATT Rev:CCAAAATCACCTCCCTCGTA | 58.3 | 410 |
| Reg13_k | Fw:GTGTCTGCTGTATGCCCTGA Rev:GAGCCAGATCTCCTGCTTTG | 59.3 | 449 |
| Reg13_l | Fw:AAATGAAGCTGGGGAGGAGT Rev:TGCATGAAGGAGTGAACAGG | 57.2 | 400 |
| Reg13_m | Fw:GAGGAAAGTGAGGTGCTTGC Rev:CTCCATTTGAGGCTGTGTGA | 58.3 | 429 |
| Reg13_n | Fw:GGCATTTGTAGCTGGGATTC Rev:CAAGCAAGGAAGGGAGTGAG | 58.3 | 426 |
| Reg13_o | Fw:AGAGATCCACCCAGTGACCA Rev:GAGTCCCGTGGATTCTACACT | 59.5 | 429 |
| Reg13_p | Fw:CTGGTGCATTTGAAAAGCTG Rev:TAAAGATGTCCGGAGGATGG | 56.2 | 434 |
| Reg13_q | Fw:GCATGGAGCTTCCTAAAGCA Rev:AGGGGGATAAGGGACATCTG | 58.3 | 407 |
| Reg13_r | Fw:TGAGACGGGAGGAGAGTTTG Rev:GTCTGGGCCTTGCTTTACAC | 59.3 | 404 |
| Reg13_s | Fw:AGGTGGAGGGGAAGGTATGT Rev:GTCCAAGAGTGGGGAACAGA | 59.3 | 427 |
| Reg13_t | Fw:CGGCCCATCTTTTAAGTCAG Rev:GCACGGGACACTACAATGTG | 58.3 | 434 |
| Reg13_u | Fw:AGCTGGTGCCTTTGAACTTG Rev:TTACATAGAGCGCCCCACAT | 57.2 | 380 |
| Reg13_v | Fw:TCTGAGACCCCCACTCCTAA Rev:GAATCCATCAGCAGTGCTCTC | 59.5 | 398 |
| Reg13_w | Fw:TCCGAGGCCTGCTTCTATTA Rev:CCTCCACACCTTTGCTGTCT | 58.3 | 353 |
| **Reg14** |  |  |  |
| Reg14_a | Fw:CTATTGAGGCACCTTGGACA Rev:GAAGTGGTGGGCTGAGAAAG | 58.3 | 569 |
| Reg14_b | Fw:GGTTCCCAAATAGGCGGTAT Rev:ACTCCCCGAAGGCAGTTTAG | 58.3 | 526 |
| Reg14_c | Fw:GAGGGGTCTGGGTGAGTCTA Rev:ATCGCACGGCTAAATCTCAG | TD:63.3🡪56.3 | 560 |
| Reg14_d | Fw:CGGTTCCAATATTTCGCAGA Rev:TCCCTCCCTATCCCTTCTTC | TD:61.3🡪54.4 | 606 |
| Reg14_e | Fw:CGAGCGTTAATAGGTTTGAGC Rev:CGCCAGGAGAGAGGAAGG | 59.2 | 588 |
| Reg14_f | Fw:CTACGGGAGAATCAGGGAGA Rev:GGTGTGTGGGGGTTGTTG | 58.9 | 535 |
| Reg14_g | Fw:GCGCTCTGCCTTTGTCTTAC Rev:CACCGCAAACTGCTTGGTAT | 58.3 | 575 |
| Reg14_h | Fw:AACGAAGTCAGCTTGCCAAC Rev:CAGGGCTGAGATCCGTCTAC | TD:63.3🡪56.3 | 583 |
| Reg14_i | Fw:CTGGTCTTGTAGGCCAGGAG Rev:TAGTGCCTGCACTGGTTCAC | 60.3 | 595 |
